# The role of the automation development group in analytical research and development at Dupont Merck

**DOI:** 10.1155/S1463924694000131

**Published:** 1994

**Authors:** John C. Lynch, Jonathan S. Green, Paul K. Hovsepian, Kathleen L. Reilly, Joseph A. Short

**Affiliations:** Analytical R & D Pharmaceutical Development The DuPont Merck Pharmaceutical Company E353 Experimental Station Wilmington Delaware 190000353 USA

## Abstract

Laboratory robotics has been firmly established in many non-QC laboratories as a valuable tool for automating pharmaceutical dosage form analysis. Often a single project or product line is used to justify an initial robot purchase thus introducing robotics to the laboratory for the first time. However, to gain widespread acceptance within the laboratory and to justify further investment in robotics, existing robots must be used to develop analyses for existing manual methods as well as new projects beyond the scope off the original purchase justification. The Automation Development Group in Analytical Research and Development is a team of analysts primarily devoted to developing new methods and adapting existing methods for the robot. This team approach developed the expertise and synergy necessary to significantly expand the contribution of robotics to automation in the authors' laboratory.


Journal of Automatic Chemistry, Vol. 16, No. 4 (July-August 1994), pp. 131-133

### The role of the automation development group in analytical research and development at Dupont Merck

**John C. Lynch, Jonathan S. Green, Paul K. Hovsepian, Kathleen L. Reilly and Joseph A. Short**
*Analytical R & D, Pharmaceutical Development The DuPont Merck Pharmaceutical Company E353 Experimental Station Wilmington, Delaware 19880-0353, USA*

*Laboratory robotics has been firmly established in many non-QC laboratories as a valuable tool for automating pharmaceutical dosage form analysis. Often a single project or product line is used to justify an initial robot purchase thus introducing robotics to the laboratory for the first time. However, to gain widespread acceptance within the laboratory and to justify further investment in robotics, existing robots must be used to develop analyses for existing manual methods as well as new projects beyond the scope off the original purchase justification. The Automation Development Group in Analytical Research and Development is a team of analysts primarily devoted to developing new methods and adapting existing methods for the robot. This team approach developed the expertise and synergy necessary to significantly expand the contribution of robotics to automation in the authors' laboratory.*

### Introduction

This paper will discuss how a small to mid-sized pharmaceutical company is successfully using automation during the early 1990s, a period of considerable and fast-paced change.

It is difficult to overemphasize how much things have changed in the research and development environment during the last 10 to 15 years. During the late 1970s and early 1980s, many research organizations experienced significant growth in their budgets for both personnel and equipment. In the areas of automation and computers, it was relatively easy to justify the purchase of new equipment if one suggested that this would help 'automate' or 'computerize' an application or function. During that same period a number of industries, including petroleum and pharmaceuticals, were enjoying a rapid increase in profits which made purchasing new equipment easier. This scenario often leads to the anecdote about the researcher who orders an expensive piece of equipment, only to leave it packed in its original box to be tripped over and eventually discarded by his replacement when the original owner is promoted or retires.

The research environment is different today. This paper will discuss the role of robotics in a pharmaceutical development organization in the midst of a changing research environment. In today's business climate, R & D costs must be not only contained but reduced. Project timelines are getting tighter; a few years ago analysts might have a year to develop methods and acquire and validate data. Now they must do that same work in at most six to nine months, with no compromise in quality. Especially in pharmaceutical R & D, there are frequent staff reorganizations which further limit the ongoing support of individuals and management for robotics, automation and new technologies in general. Automation is no longer a 'sacred cow'; it must earn its keep just like everything else in the budget by contributing to the bottom line through increases in sample throughput and productivity. Since robots have the potential to displace or change certain job functions, especially where routine sample preparation is concerned, enthusiasm among laboratory workers for robots may be severely limited in a 'downsizing' environment where everybody worries about job security. In a regulated industry like pharmaceuticals, it is necessary to constantly improve the quality and amount of the data supplied to the FDA, and with the advent of the Clinton health plan we will need to further improve cycle times if development costs are to be cut and the organization to prosper in a new era of drug cost containment.

This paper was presented at the 1993 ISLAR, organized by the Zymark Corporation.

### The role of robotics

About two years ago it was decided to bring robotics into Analytical Research and Development at DuPont Merck. Since it is relatively new company and the analytical section was mid-sized (less than 100 people) it could not support a specialized department nor could it justify the purchase of a lot of equipment. Instead robotics had to improve productivity within the constraints of the laboratory environment.

The department was responsible for developing methods and specifications for bulk drugs, dosage forms and reference standards as well as testing stability and release samples and providing for the transfer of method and technology to production sites in anticipation of NDA approval. Based on this it seemed that robotics could make the greatest impact if it could assist in the Phase II and Phase III project development.

By the time a pharmaceutical company decides to take a drug candidate into costly Phase II/III clinical trials, it has already taken a big financial gamble that it will succeed. However, even at this stage many drugs fail to show efficacy or show undesirable side-effects. Depending on the size of the clinical study, several dozen lots of various strengths, packaging and tablet shapes may be on

© Zymark Corporation 1994

stability. Stability data is not only used to establish shelf life of the various packages under different conditions (which will eventually be used in the NDA submission and in the market label) but to ensure that the various tablets which are being given to patients in the clinic meet the specifications for potency and purity. It is vitally important that samples are analysed promptly and correctly the first time to protect patients and to alert the company to possible stability and storage problems. Since samples are pulled at various intervals specified in the stability protocol for each lot, only a small time window is allowed for each analysis and its reporting. All of these factors combine for high visibility for analysts who decided to provide automated analyses to assist in these projects.

### Initial successes

During 1992 a Zymark Tablet Processing Workstation (TPW) was installed. In conjunction with the Regulatory Compliance Department and Metrologist the authors developed and carried out the validation plan, wrote and instituted metrology operating procedures, and budgeted for the training of three operators. Due to this effort over 500 sample preparations were performed during a three-month period with the TPW operators performing the HPLC analysis as well. As the original project moved toward the end of Phase III, a second TPW was purchased and installed to handle the workload anticipated as the drug moved into production and in support of other phase II/III projects. However, just as the automation effort was moving into high gear, the project was cancelled!

### Early lessons

In order to be knowledgeable about robotics, the authors learned about the technology. It is not enough to learn the basic operation for the TPW, for example it is vital to have expertise in troubleshooting, maintenance, and customizing. The authors developed partnerships with the vendor, the scientists who developed the manual methods, and with internal or external consultants.

One aspect that has deservedly received attention in several recent papers has been the importance of recruiting and developing the right staff to work with robotics. Not everyone is able to be successful in this field. People who develop and troubleshoot methods must be extremely patient but persistent. They must pay great attention to detail but still be able to see each component of the analysis as a small part of the whole. They must also be skilled analysts and have good people skills since they have to interact with many laboratory personnel who may not appreciate or understand what we do.

### Major challenges

To be successful, the authors had to overcome a number of major challenges. There was a phase II project waiting to be automated but there only was a short time—four months—in which to develop and validate the methods and issue all the required documentation. There was a wide range of strengths, and since our TPW was not equipped to do analytical dilutions, this presented a problem. There was also evidence that the solvent in the manual method caused foaming when the tablets were homogenized and that the proposed internal standard was unstable in solution. In addition, a new operator had to be trained.

This project presented another major challenge which was to the authors' advantage: the management had decided to concurrently develop a combinaton tablet as well as the single entity. Because of the similarity in products, the authors were able to use the solvent system already developed for the combination tablet manual method for both single entity and combination product automated methods. This suggestion was made by the new operator/analyst!

The purpose of this chronology is to illustrate the environment that the automation group worked in. Synergy is extremely important in an automation group. Analysts must be able to work independently but they must also communicate fully with each other and with their customers in the laboratory. There is often not enough time to develop elegant and totally novel approaches to problems. By sharing information, and using it effectively, the job gets done on time and everyone 'owns' the problem and the solution.

During 1993, in support of this project, over 400 sample preparations were performed, and three automated methods, a validation plan and report were issued and included as alternate methods in the CMC filing. This further increases our visibility and credibility. The combination product methods were issued in October 1993. However, the project chemists felt that the internal standard we were using took too long to elute. So, at their request the authors developed and validated alternate automated methods using external standard calibration. These were issued soon after the other methods, and the first samples were analysed the week before ISLAR 93.

### Path forward

With both robots busy and two of the phase III projects automated, the initial goals were met. In order to grow, the first goal was to continue to educate the department, management, and regulatory and documentation departments about the group's work. The authors also needed to continue training and developing their staff; this meant allocating funds to send them to basic and advanced Zymark training and to allow them the time to take on new tasks that would challenge them and allow them to master new skills. It was important to continue to build synergy within the group, within the department and with robotics users in other departments and sites throughout the company. There was the ongoing responsibility of managing the technology. Since both robots had been used for over a year, maintenance and troubleshooting became very important if we were to rely on them for consistent performance. Parts and supplies needed to be stocked and organized. Large quantities of reagents also needed to be stocked and stored. In addition, the authors began to work with our industrial hygiene department to monitor and limit analysts' exposure to solvents used in these analyses, as well as the noise generated by the robots. In some cases this meant making modifications to the rooms. Finally, special spill-control procedures were written and equipment ordered to minimize danger to staff and damage to equipment if any of the systems should malfunction during unattended operation. To shorten cycle times and improve quality of analyses it is necessary to constantly upgrade equipment and improve procedures, whether this is as simple as reteaching a rack position, relocating a station or working with building engineers to improve the performance of hoods or the chilled water system.

### Technology and new applications

For the longer term development of robotics in the authors' department there are two things that are important: keeping abreast of new technology and finding new niches or applications for robotics. Keeping abreast of new technology may be as simple as inviting a vendor in to demonstrate a new robot or autosampler. During 1992, the group had the opportunity to evaluate a non-Zymark workstation during a three-week period. During that time each member of our group was able to learn the rudiments of the system and prepare several samples. This experience will enable the group to comment intelligently on this vendor's product. Keeping abreast of new technology also means purchasing robots that have new or different features that may be helpful in project development. These new products may open up new applications for robotics in the lab as well as improving the automation of current ones.

The authors have found that developing new niches for robotics is a neverending task and one that is full of surprises as well. A current project challenge is to develop a robotic method for the preparation and analysis of a combination drug that is contained in a multi-layer tablet. It is a perfect application for a robot because the manual method uses large amounts of chloroform and involves a lot of repetitive motion. Using a robot can reduce the chloroform exposure and the amount of human repetitive motion. The relatively short solution stability of the product can be minimized by using the robot to prepare each sample immediately before injection. In fact, this will allow the assay for degradation products to be combined with the potency assay, saving a separate sample preparation and analysis for each sample. However, there are many challenges: the multilayer tablet is difficult to dissolve, the high rpms required for homogenization represent a noise hazard. The chloroform fumes must be contained and vented, and, finally, one of the compounds is water sensitive. To deal with these problems a Lucite containment hood has been built, which is vented to the roof scrubbers. This serves to contain the chloroform, as well as acting as a sound barrier. However, there have been problems with additional chloroform dripping from the wash line into the Fleaker during homogenization, causing dilution of the sample. In this case, the path of the tubing has been modified and a check valve added. This is a good example of how investing in new technology can help the robotics effort in unanticipated ways—the purchase was originally justified for making direct injections after sample preparation. However, several features of the robot—its smaller size, the option of using multiple solvents to wash the Fleaker rather than just water and its unique probe and Fleaker design have also helped method development in ways that were not originally foreseen.

### Conclusion

There are two lessons we have learned from our robotics group that we feel are valuable for any laboratory contemplating using robotics in an analytical research and development lab. First, as with any new technique, the more experience you have the better you become at using robotics and finding new applications. Finally, the method development process is cyclical. You return to the same issue and challenges better equipped to deal with them as well as the new challenges not previously encountered.

### Acknowledgements

The authors wish to thank K. Rick Lung, Senior Research Chemist, Mickey Schoenauer, Regulatory Compliance Manager for Information Systems, and Nancy Read, Analytical R & D's Metrologist, for their advice and help.

